# Physical interaction of STAT1 isoforms with TGF-β receptors leads to functional crosstalk between two signaling pathways in epithelial ovarian cancer

**DOI:** 10.1186/s13046-018-0773-8

**Published:** 2018-05-11

**Authors:** Xiaoling Tian, Wencai Guan, Lingyun Zhang, Wenwen Sun, Daibing Zhou, Qunbo Lin, Weimin Ren, Lubna Nadeem, Guoxiong Xu

**Affiliations:** 1 Center Laboratory, Jinshan Hospital, Fudan University, 1508 Longhang Road, Shanghai, 201508 People’s Republic of China; 2 Department of Oncology, Shanghai Medical College, Fudan University, Shanghai, 200032 China; 3 Lunenfeld Tanenbaum Research Institute, Mount Sinai Hospital, Toronto, ON M5G 1X5 Canada

**Keywords:** Activin receptor-like kinase, Cell proliferation, Crosstalk, Cytokine, Growth factor, Receptor, Signal transduction, Smad, TβRII, Tumorigenesis

## Abstract

**Background:**

The signal transducer and activator of transcription (STAT) and transforming growth factor-β (TGF-β) signaling pathways play important roles in epithelial ovarian cancer (EOC). However, the mechanism of crosstalk between two pathways is not completely understood.

**Methods:**

The expression of STAT1 protein was detected by tissue microarray and immunoblotting (IB). The interaction of STAT1 isoforms with TGF-β receptors was confirmed by immunoprecipitation and IB. The effect of TGF-β signaling on STAT1 activation was examined in EOC and non-tumorous HOSEpiC cells treated with TGF-β1 in the presence or absence of the inhibitor of TGF-β type I receptor. The gain-of-function and loss-of-function approaches were applied for detecting the role of STAT1 on EOC cell behaviours.

**Results:**

The high level of STAT1 was observed in patients with high-grade serous EOC. STAT1 expression was higher in ovarian cancer cells than noncancerous cells. TGF-β1 activated the STAT1 pathway by inducing the phosphorylation of STAT1α on S727 residue. The full-length STAT1α and the truncated STAT1β directly interacted with TGF-β receptors (ALK1/ALK5 and TβRII), which was mediated by TGF-β1. STAT1α and STAT1β blocked the activation of the TGF-β1 signaling pathway in EOC cells by reducing Smad2 phosphorylation. STAT1 overexpression induced EOC cell proliferation, migration, and invasion; whereas its inhibition enhanced TGF-β1-induced phospho-Smad2 and suppressed EOC cell proliferation, migration, and invasion.

**Conclusions:**

Our data unveil a novel insight into the molecular mechanism of crosstalk between the STAT1 and TGF-β signaling pathways, which affected the cancer cell behavior. Suppression of STAT1 may be a potential therapeutic strategy for targeting ovarian cancer.

**Electronic supplementary material:**

The online version of this article (10.1186/s13046-018-0773-8) contains supplementary material, which is available to authorized users.

## Background

Epithelial ovarian cancer (EOC) is a female malignant disease. The mechanism of the occurrence and development of EOC is complex. Cytokines and growth factors may play important roles in ovarian tumorigenesis. The signal transducer and activator of transcription 1 (STAT1) is one of the members of STAT family and functions as a signal messenger, transcription factor, and immune modulator, participating in cellular processes including cell proliferation, differentiation, apoptosis, and immunosurveillance [1–3]. STAT1 has two isoforms, a full-length STAT1α and a truncated STAT1β. STAT1α carries two phosphorylation sites, tyrosine 701 (Y701) and serine 727 (S727). The latter is located at a C-terminal trans-activation domain (TAD) [4]. STAT1β is expressed at a low level and lacks the TAD but is efficiently phosphorylated on Y701 [5, 6]. The canonical signaling pathway of STAT1 is triggered by Janus kinase (JAK) upon ligands, such as interferon-γ (IFN-γ), stimulation [7]. The phosphorylated and activated STAT1 then translocate into the nucleus and regulates the expression of target genes. For example, IFN-γ can enhance the expression of Smad7 through the JAK1/STAT1 signaling pathway [8]. Smad7 is an inhibitory Smad which prevents the interaction of Smad3 with transforming growth factor-β (TGF-β) receptor [9].

It has been shown that TGF-β plays an important role in ovarian cancer [10, 11]. The canonical TGF-β signaling pathway acts through the intracellular transducer proteins such as receptor-activated Smad (R-Smad). Upon TGF-β binding, the constitutively activated type II receptor (TβRII) recruits and phosphorylates the type I receptor (TβRI) on the cell surface [12, 13]. TβRI, also known as activin receptor-like kinase (ALK), has seven members [14]. ALK5 (TGFBRI, expressed in most types of cells) and ALK1 (ACVRL1, expressed mainly in endothelial cells) are two subtypes of TβRI for human TGF-β [15]. TGF-β1-activated ALK5 and/or ALK1 further recruit and phosphorylate R-Smads, such as Smad2/3 by ALK5 and Smad1/5/8 by ALK1. The phosphorylated and activated R-Smads then form a complex with common Smad (Co-Smad) Smad4 and the resulting complex then translocates into the nucleus where it acts as a transcription factor by binding to the promoter of a target gene to regulate its expression [16]. Smad6 and Smad7 are two inhibitory Smads (I-Smads) which prevent or inhibit Smad2/3 phosphorylation and nuclear translocation, hence suppressing their downstream function [17].

The interference between STAT1 and TGF-β signaling has been reported previously [8, 18, 19]. For instance, a bipyridyl compound CaeA can enhance TGF-β/Smad3 signaling by suppressing IFN-γ/STAT1 signaling in regulatory T cells [18]. An inhibitory action of STAT1 on TGF-β signaling is via the induction of inhibitory Smad7 [8]. On the other hand, TGF-β1 suppresses IFN-γ-induced STAT1 signaling through the promotion of STAT1 and its inhibitor protein interaction [19]. All these suggest that the crosstalk between two pathways is the downstream event of receptor activation and no exact mechanism of interference from each other is explored at the receptor level. The physical interaction of STAT1 with the signaling components of TGF-β is never speculated.

Our recent study using high-throughput luminescence-based mammalian interactome mapping technology showed that STAT1 as a potential binding protein is one of the novel interactors of the TGF-β1 receptor [20]. The current study validated for the first time that STAT1 isoforms directly interacts with TGF-β receptors and determined the consequence of this interaction particularly on the downstream signaling of TGF-β1. Meanwhile, we determined whether TGF-β1 activates STAT1 and STAT1/TGF-β receptor complex. The expression of STAT1 in EOC and the function of STAT1 on EOC cell behaviours were also examined.

## Methods

### Patients and ovarian tissue preparation

Human fresh ovarian tissue samples were obtained with informed consent from patients. The study on human subjects was approved by the Ethics Committee of Jinshan Hospital, Fudan University. A total of 20 ovarian samples were collected from patients who underwent cytoreductive surgery (5 normal samples from patients with non-ovarian tumor and 15 ovarian tumor samples, including 6 benign, 3 borderline, and 6 malignant tumors) with median age 50 years (range 25–70 years) at Jinshan Hospital from January, 2013 to January, 2016. None of the patients had received chemotherapy or radiotherapy before surgery.

### Tissue microarray and immunohistochemistry

A human ovarian tissue microarray was obtained from Alena Biotechnology Co., Ltd. (Cat# OV1005a, Xi’an, Shanxi, China). All tissues were 10% formalin-fixed and paraffin-embedded. A total of 100 ovarian tissue specimens (20 normal controls and 80 ovarian tumors) were examined by immunohistochemistry (IHC). Among 100 specimens in a slide, seven came off during the IHC staining process. In the end, 20 normal controls (3 from normal ovaries and 17 from adjacent normal ovary tissues) with median age 48.5 years (range 19–63 years) and 73 ovarian tumors (12 benign, 7 borderline, 44 malignant, 10 metastatic) with median age 49.0 years (range 17–75 years) were statistically analyzed.

After blocking with 10% normal goat serum (Fuzhou Maixin Biotech Co., Ltd., Maixin Bio, Fuzhou, Fujian, China), the sections were incubated with rabbit monoclonal antibodies against STAT1 (Cat# 9175), pSTAT1-Y701 (Cat# 9167) and pSTAT1-S727 (Cat# 8826) (Cell Signaling Technology, Inc., Danvers, MA, USA), respectively, overnight, followed by incubation with biotinylated anti-rabbit secondary antibody (Cell Signaling Technology) at room temperature for 1 h. Scoring of STAT1 immunoreactive staining was performed by two independent examiners without any prior view of patient’s clinical data and classified as described previously using staining index (SI) system [21].

### Cell culture

Human epithelial ovarian cancer cell lines (OVCAR-3 and SK-OV-3) and human embryonic kidney cell line (HEK-293 T) were obtained from American Type Culture Collection (ATCC, Manassas, VA, USA). Non-tumorous human ovarian surface epithelial cells (HOSEpiC) were obtained from ScienCell Research Laboratories (Carlsbad, CA, USA). HOSEpiC and OVCAR-3 were cultured in RPMI-1640 medium (HyClone, Thermo Fisher Scientific Inc., Beijing, China), whereas SK-OV-3 and 293 T cells were cultured in Dulbecco’s Modified Eagle’s Medium (DMEM) (HyClone), supplemented with 10% fetal bovine serum (FBS) (Invitrogen, Carlsbad, CA, USA).

### Treatment with TGF-β1 and inhibitor of TGF-β type I receptor kinase

Cells were seeded into 6-well plate at 5 × 10^5^ cells/well for 24 h and then treated with TGF-β1 (0, 0.1, 1 or 10 ng/ml, R&D Systems, Minneapolis, MN, USA) for 24 h or 10 ng/ml of TGF-β1 for a time period as indicated. In order to block the TGF-β signaling, cells were pre-treated with an inhibitor of TGF-β type I receptor kinase (10 μM SB-431542, Sigma, Saint Louis, MO, USA) for 30 min, followed by 10 ng/ml of TGF-β1 treatment for 24 h.

### Quantitative real-time polymerase chain reaction (qRT-PCR)

Total RNA was extracted from cells and tissues using the RNeasy Mini Kit (Qiagen, Gaithersburg, MD, USA) according to the manufacturer’s instruction. Five-hundred nanogram of total RNA was reverse-transcribed using a Transcriptor First Strand cDNA Synthesis Kit (Roche Diagnostics, Indianapolis, IN, USA). The primers for total STAT1, STAT1α, STAT1β, and β-actin (shown in Additional file 1: Table S1) were synthesized (GenePharma Co. Ltd., Shanghai, China). PCR amplification was performed using a Power SYBR Green PCR Master Mix Kit by 7300 Real-Time PCR System (Applied Biosystems, Foster City, CA, USA) according to the manufacturer’s recommendations.

### Transfection of small interfering RNA

Cells were seeded into 6-well plate at 2.5 × 10^5^ cells/well and transfected with 1 μg of human STAT1-small interfering RNA (STAT1-siRNA) or scramble non-specific control siRNA (NC-siRNA) (GenePharma Co. Ltd.; Additional file 1: Table S1) using X-tremeGENE siRNA transfection reagent (Roche Diagnostics) according to the manufacturer’s instruction, followed by incubation for the indicated time.

### Generation of constructs

TGF-β receptor constructs were used as described previously [20]. STAT1α and STAT1β constructs were generated by inserting the PCR products into a mammalian expression vector. Briefly, the cDNA encoding STAT1α or STAT1β was amplified by PCR using the Pfu Ultra II Fusion HS DNA Polymerase (Stratagene, Agilent Technologies, CA, USA) with specific primers (shown in Additional file 1: Table S1). After purification of PCR, the product was inserted into the *Kpn* I and *Sac* II sites of pcDNA4/TO/myc-His (B) vector (Invitrogen). Two plasmids named as pStat1α-myc and pStat1β-myc were generated and the presence of insert was confirmed by restriction enzyme digestion as well as by sequencing.

### Transient transfection and co-immunoprecipitation (co-IP)

HEK-293 T cells were seeded into 6-well plate at 2.5 × 10^5^ cells/well and were transfected or co-transfected with 4 μg receptor plasmid (ALK1-HA, ALK5-HA or TβRII-HA) and/or STAT1 plasmid (Stat1α-myc or Stat1β-myc) using DNA Transfection Reagent (GBC lifetech, Miami, FL, USA). After incubation for 48 h, cells were lysed with Pierce RIPA Buffer (Thermo Scientific, Rockford, IL, USA) supplemented with phosphatase inhibitor (KeyGEN BioTECH, Nanjing, Jiangsu, China) and PMSF (Beyotime, Haimen, Jiangsu, China) on ice for 20 min. Cell lysates (500 μg of total proteins) were then incubated with 5 μl anti-Myc IP Affinity gel or anti-HA IP Affinity gel (GBC lifetech) overnight at 4 °C according to the manufacturer’s instruction. After extensive washing, bound proteins were eluted with 4X sample buffer. Eluate and input proteins were then subjected to immunoblotting.

### Immunoblotting (IB)

Cells were lysed using Pierce RIPA buffer supplemented with 1% PMSF and phosphatase inhibitors (KeyGEN BioTECH). Protein concentration was measured using the BCA Protein Assay (Thermo Scientific). Equal amounts of protein were separated on 10% SDS-PAGE and transferred to a PVDF membrane (Millipore, Billerica, MA, USA). After blocking, the membrane was incubated with a primary antibody at 4 °C overnight and subsequently incubated with horseradish peroxidase-conjugated goat anti-rabbit or anti-mouse IgG (Cell Signaling Technology, Inc., Danvers, MA, USA) for 1 h at room temperature. The following primary antibodies were used: rabbit monoclonal anti-STAT1, anti-pSTAT1-Y701, anti-pSTAT1-S727, mouse monoclonal anti-Smad2, rabbit polyclonal anti-pSmad2, anti-β-actin (Cell Signaling Technology), rabbit polyclonal anti-HA and anti-c-Myc (Santa Cruz Biotechnology, Inc., CA, USA). Signals were detected using Immobilon™ Western Chemiluminescent HRP Substrate (Millipore) and quantified using Tanon-4500 Gel Imaging System with GIS ID Analysis Software v4.1.5 (Tanon Science and Technology Co., Ltd., Shanghai, China).

### Cell proliferation, migration, and invasion assays

For the cell proliferation assay, cells were seeded into 96-well culture plate at a density of 3 × 10^3^ cells/well and incubated for 24 h, followed by transfection with STAT1 plasmids or STAT1-siRNA or their counterpart controls in the absence or presence of 10 ng/ml of TGF-β1 for 48 h. Cell proliferation was measured by the Cell Proliferation Reagent (WST-1 kit, Roche) according to the manufacturer’s instruction. The signal was read by a microplate reader (BioTek Epoch, Winooski, VT, USA) at 450 nm.

For the migration assay, SK-OV-3 cells were seeded into 6-well plate at a density of 3 × 10^5^ cells/well and cultured for 24 h. The cell monolayer was then scraped using a pipette tip to make a scratch wound. After washing, cells were transfected with STAT1 plasmids or STAT1-siRNA as well as their counterpart controls and incubated for 24, 48, and 72 h. Cell migration was determined by wound healing. Images of the wound were obtained by photography and the gap widths were measured and analyzed.

Cell invasion was performed in a plate with a Transwell containing a porous membrane (pore size 8 μm, Costar, Corning Incorporated, New York, NY, USA) coated with Matrigel (final concentration of 250 μg/ml/well, BD Biosciences, Bedford, MA, USA). After transfection of SK-OV-3 cells with STAT1 plasmids or STAT1-siRNA or their counterpart controls for 24 h, the transfected cells were seeded on the top chamber of Transwells without serum at a density of 1 × 10^4^ cells/well. The bottom chamber was supplemented with 10% FBS as a chemoattractant. After incubation at 37 °C for 48 h, the non-invaded cells were removed by wiping the upper layer of the chamber. The invaded cells on the bottom surface were fixed with 4% paraformaldehyde and stained with 5% Crystal Violet Staining Solution (Beyotime). The cell number was counted in three random fields under a light microscope (BX43, Olympus, Tokyo, Japan). All experiments were repeated at least three times.

### Statistical analysis

Data were analyzed using GraphPad Prism 5 (GraphPad Software Inc., San Diego, CA, USA) and SPSS Statistics 21 for Windows (SPSS, Chicago, IL, USA). For comparison between two groups or multiple comparisons in treatment experiments, a Student’s t-test or one-way ANOVA was applied. Results are presented as the mean ± the standard error of the mean (SEM). The difference at *P < 0.05* was considered statistically significant.

## Results

### STAT1 expression is elevated in human high-grade serous epithelial ovarian cancer

**Fig. 1:**
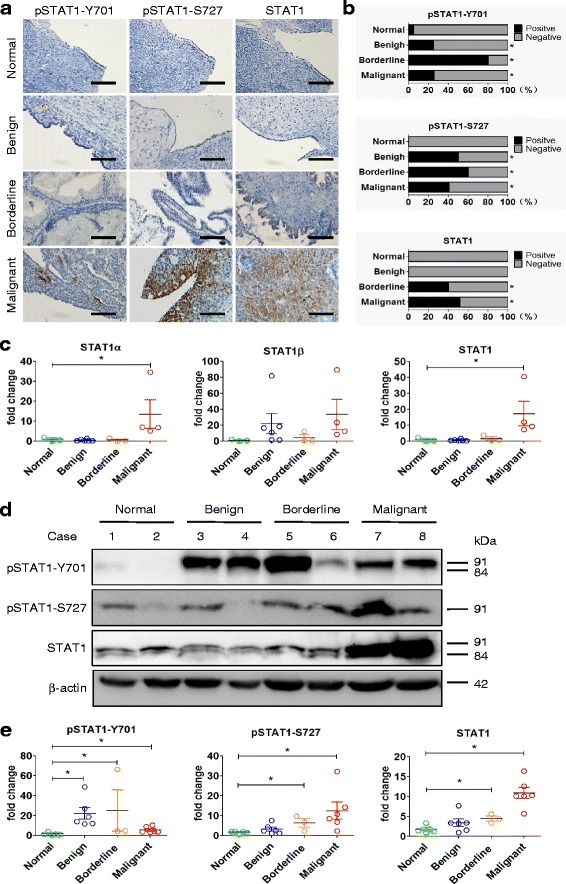
STAT1 is overexpressed in human epithelial ovarian cancer. **a** Immunohistochemical staining of STAT1 in ovarian tissues. A brown color in epithelial cells is considered as a positive staining. Representative images of pSTAT1-Y701, pSTAT1-S727, and total STAT1 expression in normal ovarian tissue (Normal), benign tumor (Benign), serous borderline tumor (Borderline), and high-grade serous carcinoma (Malignant) are shown. Original magnification, × 400; scale bar, 100 μm. **b** The case rate of pSTAT1-Y701, pSTAT1-S727, and STAT1 positivity and negativity. For comparison between two groups, χ^2^ test was applied. **c** STAT1 mRNA expression detected by quantitative RT-PCR. The expression of STAT1α and total STAT1 was higher in ovarian serous malignant tumors (Normal, *n* = 3; Benign, *n* = 6; Borderline, n = 3; Malignant, *n* = 4). **d** The expression of total STAT1, pSTAT1-Y701, and pSTAT1-S727 detected by immunoblotting. Representative images are shown. STAT1α, 91 kDa; STAT1β, 84 kDa. **e** Densitometric analysis of the gels in (**d**) (Normal, *n* = 5; Benign, n = 6; Borderline, n = 3; Malignant, n = 6). *, *P* < 0.05 compared to normal tissue

We compared the expression of STAT1 and its phosphorylated forms (pSTAT1-Y701 and pSTAT1-S727) in normal ovarian tissues and tissues of benign tumor, serous borderline tumor, and high-grade serous malignant carcinoma via immunohistochemistry. Tissue microarray showed that the positive staining of total STAT1 and phospho-STAT1 on Y701 and S727 residues in serous ovarian borderline and malignant tumors only (Fig. 1a). The tissues from borderline as well as malignant tumors showed significantly elevated levels of STAT1 as well as pSTAT1-Y701, and pSTAT1-S727 compared to the normal ovarian tissue (Fig. 1b). An array of IHC conducted on different types of ovarian cancer tissues such as serous, mucinous, endometrioid, transitional cell and metastatic tumors. We found that the expression of total STAT1, pSTAT1-Y701, and pSTAT1-S727 was elevated in all except the mucinous malignant tumors (Additional file 2: Figure S1). The comparison of the immunoreactive score between different types of the tissue showed significant differences (*P* < 0.05; Additional file 1: Table S2).

We further investigated the association of STAT1 expression with the clinicopathological features of patients with epithelial-type ovarian tumors. We found that the expression of STAT1 was not associated with the age (≤45 vs. > 45 years), histological type, lymph node metastasis, and clinical stages (*P > 0.05*; Table 1). However, when we compared the grade of serous carcinoma, a main type of EOC, we found that the total STAT1 but not the phospho-forms was significantly different between high-grade and low-grade serous carcinoma (*P = 0.031*; Table 1). The positive staining of total STAT1 was observed in all high-grade serous carcinoma (*n* = 5).

**Table 1:** Association of STAT1 Expression with Clinicopathological Features of Patients with Epithelial-Type Ovarian Tumors

Clinicopathological features		Age	pSTAT1-Y701			pSTAT1-S727			STAT1		
	n	M (Md)	+ (%)	- (%)	*P*-value	+ (%)	- (%)	P-value	+ (%)	- (%)	P-value
Age at diagnosis					0.429			0.579			0.236
≤ 45	25	39 (33)	6 (24)	19 (76)		9 (36)	16 (64)		8 (28)	17 (72)	
> 45	48	66 (53)	14 (29)	34 (71)		17 (35)	31 (65)		21 (41)	27 (59)	
Histological type of tumor					0.287^a^			0.455^b^			0.066^c^
Serous					0.065^d^			0.747^e^			0.173^f^
Benign	4	42 (42)	1 (25)	3 (75)		2 (50)	2 (50)		0 (0)	4 (100)	
Borderline	5	38 (34)	4 (80)	1 (20)		3 (60)	2 (40)		2 (40)	3 (60)	
Malignant	27	48 (50)	7 (26)	20 (74)		11 (41)	16 (59)		14 (52)	13 (48)	
Mucinous					0.333^g^			0.706^h^			0.333^i^
Benign	8	35 (32)	1 (13)	7 (87)		2 (25)	6 (75)		1 (13)	7 (87)	
Borderline	2	37 (37)	1 (50)	1 (50)		1 (50)	1 (50)		1 (50)	1 (50)	
Malignant	3	55 (63)	0 (0)	3 (100)		0 (0)	3 (100)		0 (0)	3 (100)	
Endometrioid											
Malignant	9	51 (53)	2 (22)	7 (78)		4 (44)	5 (56)		3 (33)	6 (67)	
Transitional cell											
Malignant	5	49 (51)	3 (60)	2 (40)		1 (20)	4 (80)		4 (80)	1 (20)	
LN metastasis					0.487			0.230			0.352
Yes	10	52 (54)	2 (20)	8 (80)		2 (20)	8 (80.0)		5 (50)	5 (50)	
No	63	45 (48)	17 (27)	46 (73)		24 (38)	39 (62)		24 (38)	39 (62)	
FIGO stage					0.434^j^			0.590^k^			0.156^l^
I	24	50 (52)	4 (17)	20 (83)		8 (33)	16 (67)		8 (32)	16 (68)	
II	10	50 (51)	4 (40)	6 (60)		5 (50)	5 (50)		6 (60)	4 (40)	
III	9	48 (50)	3 (33)	6 (67)		2 (22)	7 (78)		6 (67)	3 (33)	
IV	1	38 (38)	0 (0)	1 (100)		0 (0)	1 (100)		0 (0)	1 (100)	
Grade (serous)					0.072			0.274			0.031
Low-grade	29	48 (50)	5 (17)	24 (83)		10 (35)	19 (65)		13 (45)	16 (55)	
High-grade	5	52 (52)	3 (60)	2 (40)		3 (60)	2 (40)		5 (100)	0 (0)	

The expression of pSTAT1-Y701, pSTAT1-S727, and STAT1 in tissue microarray was detected by immunohistochemistry. For comparison of STAT1 protein expression associated with age, histological type, lymph node metastasis, FIGO stage and grade, Fisher’s exact test was applied. LN, lymph node; n, number of cases; M, mean; Md, median; −, negative expression; +, positive expression

^a,b,c^ Multiple comparisons of the histological types (serous, mucinous, endometrioid, and transitional cell tumors)

^d,e,f^Multiple comparisons of serous tumors (benign, borderline, and malignant tumors)

^g,h,i^Multiple comparisons of mucinous tumors (benign, borderline, and malignant tumors)

^j,k,l^Multiple comparisons of the stages

Based on the SI system, we classified STAT1 expression into positive and negative categories. Statistical analysis showed that the positivity of pSTAT1-Y701, pSTAT1-S727, and total STAT1 expression was significantly higher in ovarian borderline and malignant tumors compared with normal ovarian tissue (all *P* < 0.05; Additional file 1: Table S3).

The overexpression of STAT1 at mRNA and protein levels was further confirmed in freshly isolated ovarian tissues by qRT-PCR and immunoblotting. Using specific primers recognizing to STAT1 isoforms, we found that the mRNA of STAT1α and total STAT1 was significantly increased in ovarian malignant tumors (Fig. 1c). Immunoblotting showed that STAT1α (91 kDa) and STAT1β (84 kDa) were significantly increased in malignant tumors (Fig. 1d and e).

### STAT1α is activated by the TGF-β signaling pathway

First, we compared the endogenous expression of STAT1 between non-tumorous ovarian epithelial cell line HOSEpiC and ovarian cancer cell lines OVCAR-3 and SK-OV-3. A steady-state level of STAT1α, STAT1β, and total STAT1 mRNA was detected by qRT-PCR using specific primers (shown in Additional file 1: Table S1). We found that the expression of STAT1α, STAT1β, and total STAT1 mRNA was higher in ovarian cancer cells (OVCAR-3 and SK-OV-3) than in non-tumorous cells (HOSEpiC) (Additional file 2: Figure S2a). Similar to mRNA, the protein expression level of total STAT1 was significantly higher in OVCAR-3 and SK-OV-3 cells than in HOSEpiC cells detected by immunoblotting (Additional file 2: Figure S2b). A higher level of pSTAT1-Y701 and pSTAT1-S727 was observed in OVCAR-3 and SK-OV-3 cells, respectively, compared with HOSEpiC cells (Additional file 2: Figure S2c).

**Fig. 2:**
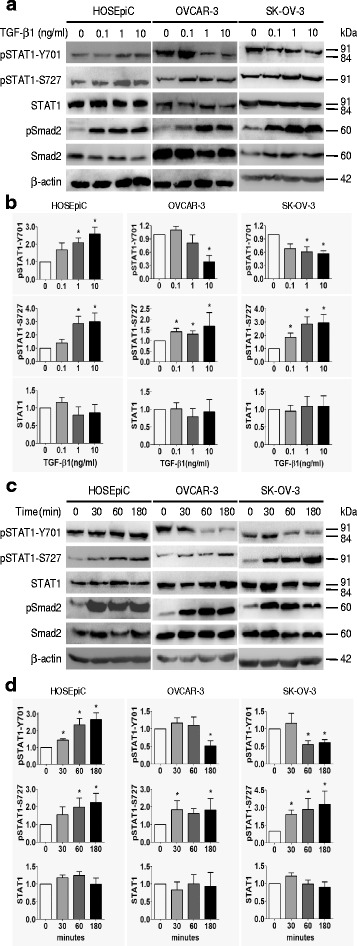
TGF-β1 regulates the phosphorylation of STAT1 in ovarian surface epithelial cells. Dose-dependent (**a**, **b**) and time-course (**c**, **d**) studies of the effect of TGF-β1 on STAT1 phosphorylation (pSTAT1) are shown. **a** Immunoblotting after cells treated with TGF-β1 (0, 0.1, 1, and 10 ng/ml) for 24 h. **b** Densitometry analysis of the gels in (**a**). **c** Immunoblotting after cells treated with TGF-β1 (10 ng/ml) for 30, 60, and 180 min. **d** Densitometric analysis of the gels in (**c**). The phosphorylation of Smad2 (pSamd2) is increased after TGF-β1 treatment, indicating the responsiveness of cells to TGF-β1. Data are represented as the mean ± SEM of the ratio of pSTAT1/total STAT1 and total STAT1/β-actin. n = 3 independent experiments; *, *P* < 0.05 compared to untreated cells

Next, we examined if TGF-β1 affects the activation status of STAT1 since in a pilot study we found that TGF-β1 increased pSTAT1-S727 (active STAT1α), while decreased pSTAT1-Y701, in OVCAR-3 cells (Additional file 2: Figure S3). In a dose-dependent study with TGF-β1 treatment for 24 h, we found that the phosphorylation of STAT1 on Y701 residue was significantly decreased (*P* < 0.05) in EOC cells (OVCAR-3 and SK-OV-3), while it was significantly increased (*P* < 0.05) in HOSEpiC cells, the non-tumorous human ovarian surface epithelial cells (Fig. 2a and b). Importantly, the phosphorylation of STAT1 on S727 residue, which represents an active form of STAT1α, was significantly increased (*P* < 0.05) in all three epithelial-type cell lines after TGF-β1 treatment (Fig. 2a and b). In a time-course study with a constant dose (10 ng/ml) of TGF-β1, we also observed that the phosphorylation of STAT1 on Y701 was significantly increased (*P* < 0.05) in HOSEpiC cells, but decreased (*P* < 0.05) in OVCAR-3 and SK-OV-3 cells, and that on S727 was significantly increased (*P* < 0.05) in all three cell lines (Fig. 2c and d). TGF-β1 did not affect total-STAT1 in all investigated ovarian surface epithelial cells. Phosphorylation of Smad2 was used to validate the activation of TGF-β signaling upon stimulation with TGF-β1 and an increase in phospho-Smad2 after TGF-β1 treatment indicates the responsiveness of these cells to TGF-β1.

**Fig. 3:**
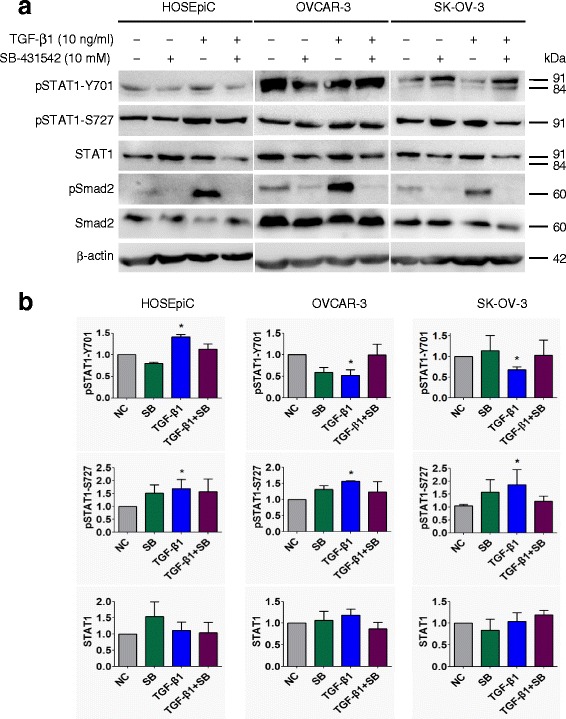
Inhibition of TGF-β receptor kinase blocks the effect of TGF-β on STAT1 phosphorylation in ovarian surface epithelial cells. Cells were pretreated with 10 μM TβRI inhibitor SB-431542 for 30 min, followed by 10 ng/ml of TGF-β1 treatment for 24 h. **a** Immunoblotting after cells treated with SB-431542 and/or TGF-β1. **b** Densitometric analysis of the gels in (**a**). The responsiveness of cells to TGF-β1 was confirmed by the detection of pSmad2. Data are represented as the mean ± SEM of the ratio of pSTAT1 over total STAT1 or total STAT1 over β-actin. NC, non-treated control; SB, SB-431542; *n* = 3 independent experiments; *, *P* < 0.05 compared to NC

Subsequently, we verified our results with an alternate strategy by blocking TGF-β signaling in ovarian surface epithelial cells with a TβRI specific inhibitor SB-431542. Cells were pretreated with 10 μM SB-431542 for 30 min, followed by treatment with 10 ng/ml TGF-β1 for 24 h. The inhibitor treatment clearly and significantly (*P* < 0.05) abolished the effects of TGF-β1 on STAT1 phosphorylation on Y701 and S727 sites (Fig. 3a and b). Thus, our data demonstrated that the phosphorylation and activation of STAT1 can be mediated by the TGF-β signaling pathway and may be a consequence of the direct interaction of STAT1 with TGF-β receptors after TGF-β1 stimulation.

### STAT1α and STAT1β interact with TGF-β type I and type II receptors

**Fig. 4:**
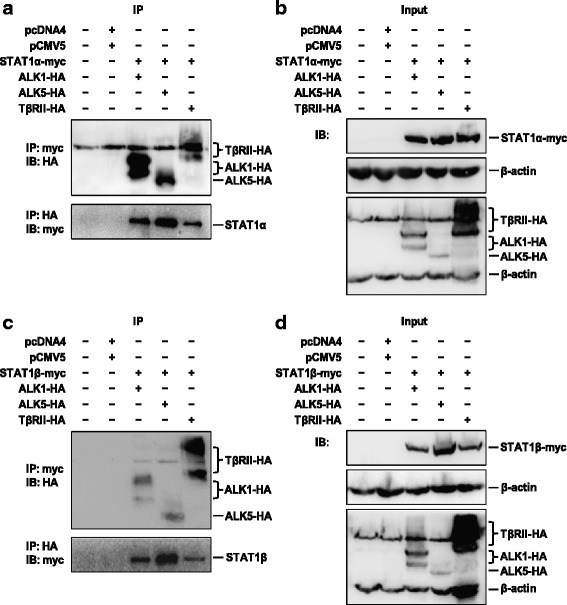
STAT1 interacts with the TGF-β1 receptor. HEK-293 T cells were transiently co-transfected with STAT1α-myc or STAT1β-myc and ALK1-HA, ALK5-HA or TβRII-HA plasmids and incubated for 48 h. **a** Interaction of STAT1α with receptors detected by immunoprecipitation (IP) with anti-myc antibody for STAT1α, followed by immunoblotting (IB) with anti-HA antibody for receptors (upper panel), or IP with anti-HA antibody for receptors, followed by IB with anti-myc antibody for STAT1α (bottom panel). **b** IB using antibodies specific to β-actin, myc for STAT1α, and HA for receptors. **c** Interaction of STAT1β with receptors detected by IP with anti-myc antibody for STAT1β, followed by IB with anti-HA antibody for receptors (upper panel), or vice versa (bottom panel). **d** IB using antibodies specific to β-actin, myc for STAT1β, and HA for receptors. pcDNA4 and pCMV5 are two empty vectors used as negative controls. Each experiment is repeated at least once. Representative images are shown. STAT1α-myc, 93 kDa; STAT1β-myc, 86 kDa; ALK1-HA, 58–69 kDa; ALK5-HA, 53 kDa; TβRII-HA, 71–80 kDa

In order to assess STAT1α/β and TGF-β receptor interaction in vitro, we used HEK-293 T cells and transiently transfected them with myc-tagged STAT1α/STAT1β and co-transfected them with HA-tagged ALK1/ALK5/TβRII plasmid vectors. Co-IP was performed with anti-myc or anti-HA antibody. The bound TGF-β receptors or STAT1 isoforms were detected with anti-HA or anti-myc IB respectively. We found that both STAT1α and STAT1β interact with ALK1, ALK5, and TβRII receptors (Fig. 4a and b). The overexpression of STAT1α, STAT1β, and receptor proteins was validated by IB (Fig. 4c and d).

### Interaction of STAT1 with TGF-β receptors is mediated by TGF-β1

**Fig. 5:**
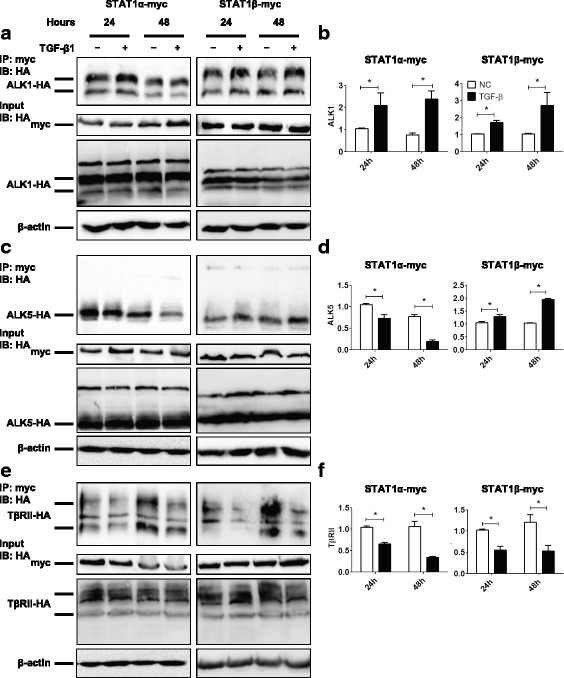
TGF-β1 regulates the interaction of STAT1α/STAT1β with ALK1/ALK5/TβRII. HEK-293 T cells were transiently co-transfected with STAT1α-myc or STAT1β-myc and ALK1-HA, ALK5-HA or TβRII-HA plasmids in the absence or presence of TGF-β1 (10 ng/ml) for 24 and 48 h. **a** Immunoprecipitation (IP) with anti-myc antibody for STAT1α-myc or STAT1β-myc and immunoblotting (IB) with anti-HA antibody for ALK1-HA. Cell lysates of input samples were used for detecting the expression of STAT1-myc, ALK1-HA, and β-actin. **b** Densitometric and semi-quantitative analyses of the gels of IP in (**a**). **c** IP with anti-myc antibody for STAT1α-myc or STAT1β-myc and IB with anti-HA antibody for ALK5-HA. Cell lysates of input samples were used for detecting the expression of STAT1-myc, ALK5-HA, and β-actin. **d** Densitometric and semi-quantitative analyses of the gels of IP in (**c**). **e** IP with anti-myc antibody for STAT1α-myc or STAT1β-myc and IB with anti-HA antibody for TβRII-HA. Cell lysates of input samples were used for detecting the expression of STAT1-myc, TβRII-HA, and β-actin. **f** Densitometric and semi-quantitative analyses of the gels of IP in (**e**). *n* = 3 independent experiments; *, *P* < 0.05

To determine whether TGF-β1 affects the complex of STAT1 and TGF-β receptors, HEK-293 T cells were co-transfected with STAT1α or STAT1β and ALK1, ALK5, or TβRII plasmids, followed by 10 ng/ml of TGF-β1 treatment. Co-IP assay revealed that TGF-β1 enhanced STAT1α and STAT1β interaction with ALK1 (Fig. 5a and b). Interestingly, TGF-β1 suppressed STAT1α, while enhanced STAT1β, interaction with ALK5, the main type I receptor of TGF-β (Fig. 5c and d). Furthermore, TGF-β1 suppressed STAT1α and STAT1β interaction with TβRII (Fig. 5e and f). These data indicate that TGF-β1 may mediate the association and dissociation of STAT1α/β with TβRI/TβRII receptors.

### STAT1 inhibits the TGF-β signaling pathway

**Fig. 6:**
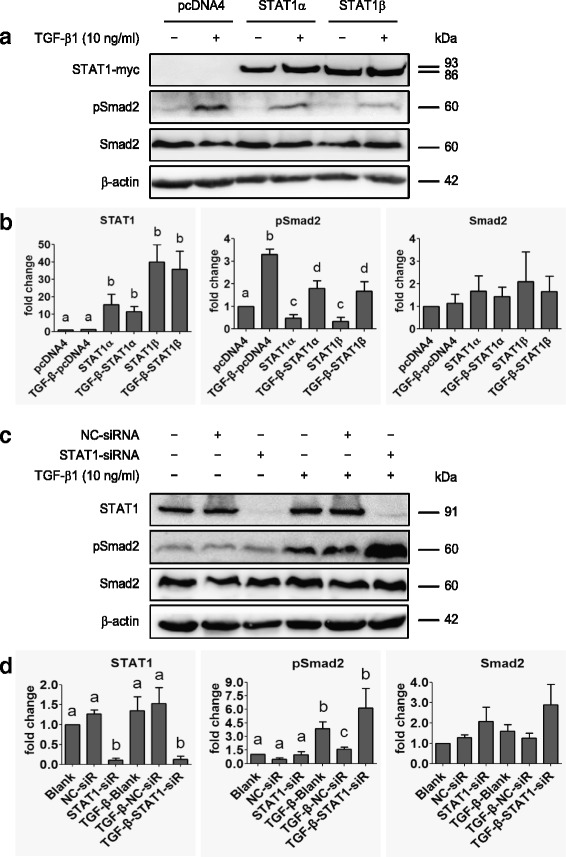
STAT1 affects Smad2 phosphorylation. **a** Immunoblotting after SK-OV-3 cells transiently transfected with pcDNA4, STAT1α or STAT1β plasmid in the absence or presence of TGF-β1 (10 ng/ml) for 24 h. **b** Densitometric analysis of the gels in (**a**). **c** Immunoblotting of STAT1 and Smad2 in STAT1-knockdown SK-OV-3 cells in the absence or presence of TGF-β1 (10 ng/ml) for 24 h. **d** Densitometric analysis of the gels in (**c**). Data are represented as the mean ± SEM of the ratio of pSmad2/total Smad2, total Smad2/β-actin, and total STAT1/β-actin. Different superscript denotes statistically significant difference (*P* < 0.05; n = 3 independent experiments)

In order to examine the significance of STAT1 interaction with TGF-β receptors, we performed overexpression or knockdown of STAT1α/β in ovarian cancer cells (SK-OV-3) and examined the status of the TGF-β signaling pathway. We found that pSmad2 was increased after 10 ng/ml TGF-β1 treatment for 24 h, indicating that the TGF-β signaling pathway was intact in SK-OV-3 cells (Fig. 6a and b). However, a significant inhibition of Smad2 phosphorylation was observed in STAT1α or STAT1β expressing cells even when the cells were stimulated with TGF-β1 (Fig. 6a and b). On the other hand, knockdown of endogenous STAT1 in SK-OV-3 cells using STAT1-siRNA resulted in a significant increase of TGF-β1-induced Smad2 phosphorylation (Fig. 6c and d). These data indicate that STAT1 inhibits the TGF-β signaling pathway.

### STAT1 promotes EOC cell proliferation, migration, and invasion

**Fig. 7:**
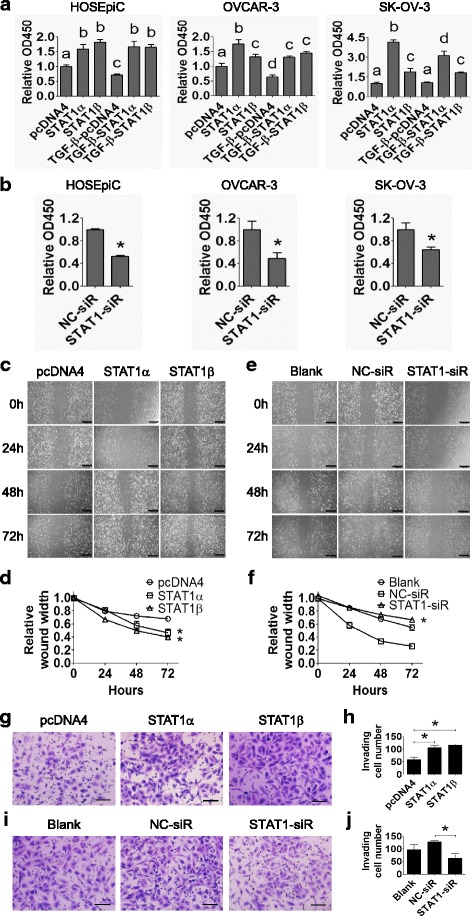
STAT1 increases ovarian surface epithelial cell proliferation, migration, and invasion. **a** HOSEpiC, OVCAR-3, and SK-OV-3 cells were transiently transfected with STAT1α or STAT1β plasmid in the absence or presence of TGF-β1 (10 ng/ml) for 48 h. Cell proliferation was measured by the WST-1 assay. pcDNA4 was used as negative control. Different superscript denotes statistically significant difference (*P* < 0.05; n = 3 independent experiments). **b** HOSEpiC, OVCAR-3, and SK-OV-3 cells were transiently transfected with STAT1-siRNA or non-specific control (NC)-siRNA. Cell proliferation was measured by the WST-1 assay at 48 h post-transfection (*, *P* < 0.05; n = 3 independent experiments). **c** Wound healing assay in SK-OV-3 cells after transiently transfecting with STAT1α or STAT1β plasmid for 24, 48, and 72 h. **d** Quantitative analysis of the wound width in (**c**). **e** Wound healing assay in SK-OV-3 cells after transiently transfecting with STAT1-siRNA (STAT1-siR) for 24, 48, and 72 h. **f** Quantitative analysis of the wound width in (**e**). STAT1 promotes the migration of SK-OV-3 cells. Original magnification, × 200; scale bars, 500 μm. **g** Invasion assay of SK-OV-3 cells after transiently transfecting with STAT1α or STAT1β plasmid for 48 h. **h** Quantitative analysis of invaded cells in (**g**). **i** Invasion assay of SK-OV-3 cells after transiently transfecting with STAT1-siRNA or NC-siRNA for 48 h. **j** Quantification analysis of invaded cells in (**i**). Invaded cells were counted from three random fields. STAT1 promotes the invasion of SK-OV-3 cells. Original magnification, × 200; scale bar, 500 μm. Data are presented as the mean ± standard deviation (SD). *, *P* < 0.05 compared to control

To investigate the role of STAT1 on cell behavior in ovarian cancer cells, the gain-of-function and loss-of-function approaches were applied. STAT1α or STAT1β was overexpressed using STAT1α-myc or STAT1β-myc plasmids in HOSEpiC, OVCAR-3 and SK-OV-3 cells in the presence or absence of 10 ng/ml of TGF-β1 for 48 h. Cell proliferation was analyzed with WST-1 assay which showed that both STAT1α and STAT1β significantly increased cell proliferation in all investigated cell lines (*P* < 0.05), while TGF-β1 inhibits it in HOSEpiC and OVCAR-3 cells but not in SK-OV-3 cells (Fig. 7a). Importantly, TGF-β1 failed to inhibit cell proliferation in STAT1α or STAT1β overexpressing HOSEpiC cells and OVCAR-3 cells (Fig. 7a). On the other hand, the siRNA mediated knockdown of endogenous STAT1 resulted in a decrease of cell proliferation in all three cell lines (Fig. 7b). These data suggest that STAT1 promotes ovarian surface epithelial cell proliferation and blocks the inhibitory effect of TGF-β on cell proliferation.

Using the same strategy of STAT1 overexpression and knockdown we assessed the effect of STAT1α and STAT1β on SK-OV-3 cell migration using wound healing and cell invasion using Matrigel-transwell invasion assays. Overexpression of STAT1α and STAT1β significantly increased (*P* < 0.05), and the suppression of STAT1 significantly decreased (*P* < 0.05), cell migration (Fig. 7c-f) and invasion (Fig. 7g-j) compared with their respective controls.

## Discussion

The current study demonstrated for the first time that STAT1α and STAT1β directly interact with TGF-β receptors (ALK1/ALK5/TβRII) and that the phosphorylation of STAT1 on Y701 and S727 is mediated by TGF-β1. Furthermore, the overexpression or knockdown of STAT1 influences TGF-β1-induced Smad2 phosphorylation and affects EOC cell proliferation, migration, and invasion. We convince that the crosstalk between two pathways initiates at the receptor level.

STAT1 is a transcriptional factor which mediates responses to all types of IFNs and regulates a variety of cellular activities [22], whereas the impairment of TGF-β signaling has been found in various diseases, including cancer [23]. Although the interference between STAT1 and TGF-β signaling has been reported previously, all data indicate that the crosstalk between these two pathways is the downstream event of receptor activation. Furthermore, no exact mechanism of interference from each other is explored and the physical interaction of STAT1 with the signaling components of TGF-β is never hypothesized. Using a high-throughput luminescence-based mammalian interactome mapping technology we have recently reported that STAT1 might interact with the TGF-β type I and type II receptors [20]. The present study verified that STAT1α/β indeed bind to TβRII/TβRI (ALK1 and ALK5) and negatively regulates TGF-β signaling.

We consistently observed that TGF-β1 induced STAT1 phosphorylation on Y701 and S727 in non-tumorous cells. In cancerous cells, however, TGF-β1 induced STAT1 phosphorylation on S727 only (specific to STAT1α form) and inhibits STAT1 phosphorylation on Y701 (specific to both STAT1α and STAT1β forms). These data suggest that, besides IFNs, TGF-β1 also modulates the STAT1 signaling pathway. Interestingly, the binding status of STAT1α and STAT1β with ALK5, the main type I receptor of TGF-β, was different between non-cancerous and cancerous cells upon the administration of a ligand TGF-β1. It might depend on the certain circumstances. We hypothesize that there is a homeostasis function of STAT1α and STAT1β in the normal situation, which TGF-β1 activates both sites (Y701 and S727); whereas there is abnormal higher level of STAT1 in a tumor cell, which TGF-β1 increases the phosphorylation of STAT1 at S727 site (STAT1α) and decreases the phosphorylation of STAT1 at Y701 site (STAT1α/β). TGF-β-mediated STAT1 phosphorylation and activation may be cell-type specific and may reflect a molecular shift in tumorigenesis. Similar to our finding, it has been shown that TGF-β inhibits the activation and phosphorylation of STAT1 on Y701 induced by insulin-like growth factor binding protein-3 (IGFBP-3) in mesenchymal chondroprogenitor cells [24]. TGF-β1 also inhibits IFN-γ-induced phosphorylation of STAT1 on Y701 and S727 in glial cells from rat brain [25]. However, none of these studies show that the phosphorylation of STAT1 is a consequence of its binding to the TGF-β receptor. The current study provides conceivable evidence that an increase of STAT1 phosphorylation on S727, similar to Smad2 phosphorylation, can be detected within a short time (30 min) upon TGF-β1 stimulation, indicating that this process is associated with TGF-β receptor binding after the cytokine treatment.

Our study has shown that the status of the association or dissociation of STAT1/TβRI/TβRII complex is altered after TGF-β1 treatment. Classically, the activated STAT1 should dissociate from the TβRII/ALK5 complex, dimerize and translocate to the nucleus. However based on our co-immunoprecipitation data we speculate that the interaction between STAT1α and TGF-β receptor is not transient and that STAT1α constitutively binds to the TGF-β receptor, and blocks Smad phosphorylation and hence the downstream TGF-β signaling pathway. The phosphorylation of STAT1α by TGF-β1 leads to its activation and the dissociation of STAT1α from TβRII/ALK5 receptor complex that releases the blockage and, in turn, increases the phosphorylation of Smad2, executing TGF-β signal transduction in ovarian cancer cells. Furthermore, the balance between STAT1α and STAT1β is important in ovarian tumorigenesis. It has been showed the overexpression of STAT1β can inhibit the phosphorylation of STAT1α as well as the DNA-binding and transcriptional activities in B lymphocytes [26], indicating that the altered levels of the STAT1 isoforms may affect the pathophysiological processes. In support of our data that STAT^−/−^ mice had high activation of the TGF-β signaling pathway during liver fibrosis [27], knockdown of STAT1 enhances TGF-β1-induced phospho-Smad2, whereas overexpression of STAT1 suppresses TGF-β1-induced phospho-Smad2, strongly pointing toward the influence of STAT1 on TGF-β signaling pathway.

With respect to tumorigenesis both STAT1 and TGF-β1 present controversial roles. STAT1 has been reported for its tumor suppressive as well as tumor promoting functions [28], whereas TGF-β inhibits cell proliferation at an early stage and promotes invasion and metastasis at the later stage of cancer [29]. TGF-β-mediated STAT1 activation via STAT1α phosphorylation may result in the promotion of tumorigenesis. The current study showed that the expression level of STAT1 was higher in ovarian cancer cells (OVCAR-3 and SK-OV-3) than non-cancerous ovarian cells (HOSEpiC). High level of STAT1 was also observed in patients with high-grade serous EOC. The overexpression of STAT1 in ovarian cancer may result in the tumorigenic effect of TGF-β signaling and therefore partially explains the controversial behavior of TGF-β in tumorigenesis. Similar to the results reported previously in endometrial cancer cells [30], our study demonstrated that the elevation of STAT1 expression promotes while its knockdown inhibits EOC cell proliferation, migration, and invasion.

In the present study, we found overexpression of STAT1 at both mRNA and protein levels in human epithelial-type ovarian borderline and malignant tumor tissues. High level of STAT1 was found in ovarian serous malignant tumors rather than in mucinous tumors, indicating that it is a tissue biomarker at least and is tumor-type specific. STAT1 has been recently identified as a drug resistance biomarker in ovarian cancer [31]. It has been reported that STAT1 is a potential indicator predicting chemoresistance in EOC [32, 33]. Activating the FAK/STAT1 signaling pathway induces a malignant potential in ovarian epithelium [34] and targeting this signaling pathway is a good therapeutic strategy for ovarian cancer [35].

## Conclusions

**Fig. 8:**
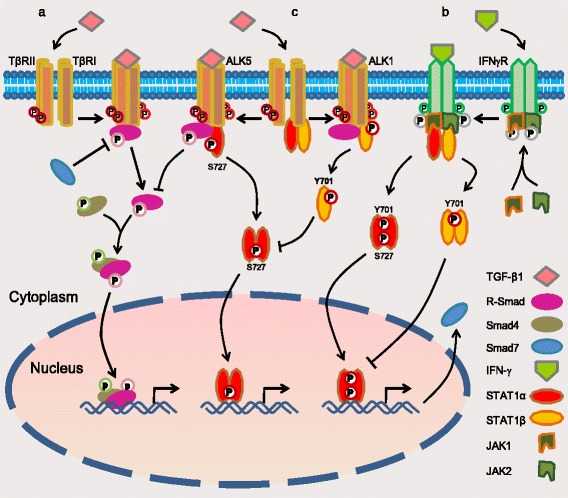
A schematic of the model of crosstalk between STAT1 and TGF-β signaling pathways. **a** The canonical signaling pathway of TGF-β: upon TGF-β1 binding to type II receptor (TβRII), the activated TβRII recruits and activates type I receptor (TβRI: ALK5 in most type of cells or ALK1 in endothelial cells). The activated receptor kinases then phosphorylate R-Smads such as Smad2/3 (by ALK5) and Smad1/5/8 (by ALK1). Activated R-Smads form the complex with Smad4 and translocate to the nucleus where they regulate target genes [12, 36]. **b** The canonical signaling pathway of STAT1: upon IFN-γ binding to its receptor, JAKs are phosphorylated and activated each other. The activated JAKs along with the intracellular tail of receptor recruit and activate STAT1 by the phosphorylation of STAT1 on Y701 and/or S727, which promotes them to dimerize and enter the nucleus where they regulate gene expression [7, 37], e.g. gene *Smad7*. Protein Smad7 prevents R-Smad interaction with TGF-β receptor [8]. Overexpression of STAT1β inhibits STAT1α activity to maintain the balance between two isoforms [26]. **c** The crosstalk of the STAT1 and TGF-β signaling pathways: STAT1 constitutively interacts with TβRII/TβRI (either ALK5 or ALK1 based on cell type). Upon TGF-β1 binding to TβRII/ALK5, the receptor-complex increases the phosphorylation of STAT1 on S727 (the active form of STAT1α). The activated STAT1 then dissociates itself from TβRII/ALK5 complex and executes its mission of transcription factor. On the other hand, STAT1 protein is increased in ovarian cancer cells and binds to TGF-β receptors. Overexpressed STAT1 suppresses TGF-β-induced Smad2 phosphorylation and blocks, at least in part, the TGF-β signaling pathway (current work)

STAT1 is a tissue biomarker of serous-type EOC. Overexpression of STAT1 inhibits, whereas a decrease of STAT1 enhances, TGF-β-mediated suppression of cell proliferation, migration, and invasion. STAT1 directly interacts with TβRII/TβRI (either ALK5 or ALK1 based on cell type) and is activated by TGF-β1, while TGF-β signaling is blocked by STAT1 as a consequence of this interaction (Fig. 8). These data unveil a new insight into the molecular mechanisms of crosstalk between the STAT1 and TGF-β signaling pathways and suggest that STAT1 is a potential therapeutic target for EOC treatment.

## Additional files

Additional file 1: **Table S1.** PCR primer and siRNA sequence used in experiments. **Table S2.** Comparison of pSTAT1-Y701, pSTAT1-S727, and STAT1 immunostaining in the ovarian tissues. **Table S3.** The expression of pSTAT1-Y701, pSTAT1-S727, and STAT1 in human ovarian tissues. (DOCX 50 kb)

Additional file 2: **Figure S1.** STAT1 expression in human epithelial-type ovarian tumors. Tissue microarray shows the immunohistochemical (IHC) staining of pSTAT1-Y701, pSTAT1-S727, and total STAT1 in serous, mucinous, endometrioid, transitional cell, and metastatic tumors. **Figure S2.** STAT1 expression in ovarian surface epithelial cells. a STAT1 mRNA expression detected by quantitative RT-PCR. b STAT1 protein expression detected by immunoblotting. c Densitometric analysis of the gels. **Figure S3.** Effect of TGF-β1 on the phosphorylation of STAT1. (DOCX 1304 kb)

## Abbreviations

ALK: Activin receptor-like kinase
IFN-γ: Interferon-γ
JAK: Janus kinase
siRNA: small interfering RNA
STAT: Signal transducer and activator of transcription
TGF-β: Transforming growth factor-β
TβRI: TGF-β type I receptor
TβRII: TGF-β type II receptor
