# Supplementary material for: Physical interaction of STAT1 isoforms with TGF-β receptors leads to functional crosstalk between two signaling pathways in epithelial ovarian cancer
Source: J Exp Clin Cancer Res. 2018 May 11;37:103. doi: 10.1186/s13046-018-0773-8 (PMC5948853; doi:10.1186/s13046-018-0773-8)
Supplement: Supplementary file 1 — Table S1. PCR primer and siRNA sequence used in experiments. Table S2. Comparison of pSTAT1-Y701, pSTAT1-S727, and STAT1 immunostaining in the ovarian tissues. Table S3. The expression of pSTAT1-Y701, pSTAT1-S727, and STAT1 in human ovarian tissues. (DOCX 50 kb) [file 13046_2018_773_MOESM1_ESM.docx]

**Additional file 2:**

# Table S1. PCR primer and siRNA sequence used in experiments

| Target primer and siRNA | **Sequence**  **5’ → 3’** | **Position in sequence** | Product size (bp) | GenBank accession # |
| --- | --- | --- | --- | --- |
| Construct primer |  |  |  |  |
| pStat1α-myc |  |  |  |  |
| Forward | ggggtacccGGCAGGATGTCTCAGTGGTACGAACTTC | nt 383-410 | 2282 | NM_007315 |
| Reverse | cgggatccctaccgcggTACTGTGTTCATCATACTGTC | nt 2618-2638 |  |  |
| pStat1β-myc |  |  |  |  |
| Forward | ggggtacccGGCAGGATGTCTCAGTGGTACGAACTTC | nt 383-410 | 2168 | NM_139266 |
| Reverse | cgggatccattccgcggCACTTCAGACACAGAAATCAAC | nt 2503-2524 |  |  |
| PCR primer |  |  |  |  |
| STAT1 (total) |  |  |  |  |
| Forward | TCCGTTTTCATGACCTCCTG | nt 552-571 | 229 | NM_007315 |
| Reverse | TGAATATTCCCCGACTGAGC | nt 761-780 |  |  |
| STAT1α |  |  |  |  |
| Forward | TGATGGCCCTAAAGGAACTG | nt 2467-2486 | 234 | NM_007315 |
| Reverse | GAGGGAATCACAGATGAGAAG | nt 2680-2700 |  |  |
| STAT1β |  |  |  |  |
| Forward | TGATGGCCCTAAAGGAACTG | nt 2467-2486 | 105 | NM_139266 |
| Reverse | AGGCTGGCTTGAGGTTTGTA | nt 2552-2571 |  |  |
| β-actin |  |  |  |  |
| Forward | CATTGCCGACAGGATGCAG | nt 1008-1026 | 169 | NM_001101 |
| Reverse | CTCGTCATACTCCTGCTTGCTG | nt 1155-1176 |  |  |
| siRNA |  |  |  |  |
| STAT1-siRNA |  |  |  |  |
| Sense | GCGUAAUCUUCAGGAUAAUtt | nt 649-667 |  | NM_007315 |
| Antisense | AUUAUCCUGAAGAUUACGCtt |  |  |  |
| Control-siRNA |  |  |  |  |
| Sense | UUCUCCGAACGUGUCACGUtt |  |  |  |
| Antisense | ACGUGACACGUUCGGAGAAtt |  |  |  |

# The low case indicates a linker. Underline indicates a restriction enzyme *Kpn* I or *Sac* II. bp, base pair; nt, nucleotide

**Table S2.** Comparison of pSTAT1-Y701, pSTAT1-S727, and STAT1 immunostaining in the ovarian tissues

|  | pSTAT1-Y701 | | pSTAT1-S727 | | | STAT1 | | |
| --- | --- | --- | --- | --- | --- | --- | --- | --- |
| Comparison | Z score | P-value | | Z score | P-value | | Z score | P-value |
| Normal *vs.* Benign | -1.000 | 0.540 | | -2.717 | 0.014 | | -1.291 | 0.375 |
| Normal *vs.* Borderline | -2.826 | 0.005 | | -3.580 | 0.000 | | -3.043 | 0.002 |
| Normal *vs.* Malignant | -1.906 | 0.082 | | -3.014 | 0.003 | | -3.534 | 0.000 |
| Normal *vs.* Metastasis | -1.183 | 0.237 | | -2.035 | 0.042 | | -2.978 | 0.003 |
| Benign *vs.* Borderline | -2.044 | 0.045 | | -1.703 | 0.101 | | -1.783 | 0.090 |
| Benign *vs.* Malignant | -0.609 | 0.707 | | -0.563 | 0.612 | | -2.387 | 0.019 |
| Benign *vs.* Metastasis | -0.098 | 0.789 | | -0.338 | 0.717 | | -1.842 | 0.078 |
| Borderline *vs.* Malignant | -1.685 | 1.000 | | -1.747 | 0.072 | | -0.226 | 0.838 |
| Borderline *vs.* Metastasis | -1.530 | 0.126 | | -1.604 | 0.109 | | -0.110 | 0.913 |
| Malignant *vs.* Metastasis | -0.497 | 0.675 | | -0.639 | 0.554 | | -0.074 | 0.951 |

Wilcoxon rank-sum test was used to analyze pSTAT1-Y701, pSTAT1-S727, and STAT1 immunoreactive scores between two types of tissue

**Table S3.** The expression of pSTAT1-Y701, pSTAT1-S727, and STAT1 in human ovarian tissues

|  |  | Age | pSTAT1-Y701 | | | pSTAT1-S727 | | | STAT1 | | |
| --- | --- | --- | --- | --- | --- | --- | --- | --- | --- | --- | --- |
|  | n | Mean (Median) | + | - | % | + | - | % | + | - | % |
| Normal | 20 | 45 (48) | 1 | 19 | 5 | 0 | 20 | 0 | 0 | 20 | 0 |
| Benign | 12 | 37 (36) | 2 | 10 | 17 | 4 | 8 | 33 | 1 | 11 | 8 |
| Borderline | 7 | 38 (34) | 5 | 2 | 71 | 4 | 3 | 57 | 3 | 4 | 43 |
| Malignant | 44 | 49 (51) | 12 | 32 | 27 | 16 | 28 | 36 | 20 | 24 | 46 |
| Metastasis | 10 | 46 (49) | 2 | 8 | 20 | 2 | 7 | 22 | 5 | 5 | 50 |

Based on the SI system, the positivity and negativity categories were classified. For comparison between 2 groups, a Fisher's exact test was applied

n, number of cases; normal, normal ovarian tissue; benign, ovarian benign tumor; borderline, ovarian borderline tumor; malignant, ovarian malignant tumor; Metastasis, metastatic adenocarcinoma. +, positive staining; -, negative staining; %, positive rate

Statistical analysis:

pSTAT1-Y701: normal *vs.* benign, P=0.310; normal *vs.* borderline, P=0.001; normal *vs.* malignant, P<0.05; normal *vs*. metastasis, P=0.251; benign *vs*. borderline, P<0.05; benign *vs*. malignant, P=0.367; benign *vs*. metastasis, P=0.632; borderline *vs.* malignant, P<0.05; borderline *vs*. metastasis, P=0.052; malignant *vs*. metastasis, P=0.488

pSTAT1-S727: normal *vs.* benign, P<0.05; normal *vs.* borderline, P<0.05; normal *vs.* malignant, P=0.001; normal *vs*. metastasis, P=0.089; benign *vs.* borderline, P=0.297; benign *vs.* malignant, P=0.565; benign *vs*. metastasis, P=0.477; borderline *vs.* malignant, P=0.247; borderline *vs*. metastasis, P=0.182; malignant *vs*. metastasis, P=0.344

STAT1: normal *vs.* benign, P=0.375; normal *vs.* borderline, P<0.05; normal *vs.* malignant, P<0.001; normal *vs*. metastasis, P<0.01; benign *vs.* borderline, P=0.117; benign *vs.* malignant, P=0.02; benign *vs*. metastasis, P<0.05; borderline *vs.* malignant, P=0.613; borderline *vs*. metastasis, P=0.581; malignant *vs*. metastasis, P=0.534
