# Supplementary material for: Physical interaction of STAT1 isoforms with TGF-β receptors leads to functional crosstalk between two signaling pathways in epithelial ovarian cancer
Source: J Exp Clin Cancer Res. 2018 May 11;37:103. doi: 10.1186/s13046-018-0773-8 (PMC5948853; doi:10.1186/s13046-018-0773-8)
Supplement: Supplementary file 2 — Figure S1. STAT1 expression in human epithelial-type ovarian tumors. Tissue microarray shows the immunohistochemical (IHC) staining of pSTAT1-Y701, pSTAT1-S727, and total STAT1 in serous, mucinous, endometrioid, transitional cell, and metastatic tumors. Figure S2. STAT1 expression in ovarian surface epithelial cells. a STAT1 mRNA expression detected by quantitative RT-PCR. b STAT1 protein expression detected by immunoblotting. c Densitometric analysis of the gels. Figure S3. Effect of TGF-β1 on the phosphorylation of STAT1. (DOCX 1304 kb) [file 13046_2018_773_MOESM2_ESM.docx]

**Additional file 1:**

**
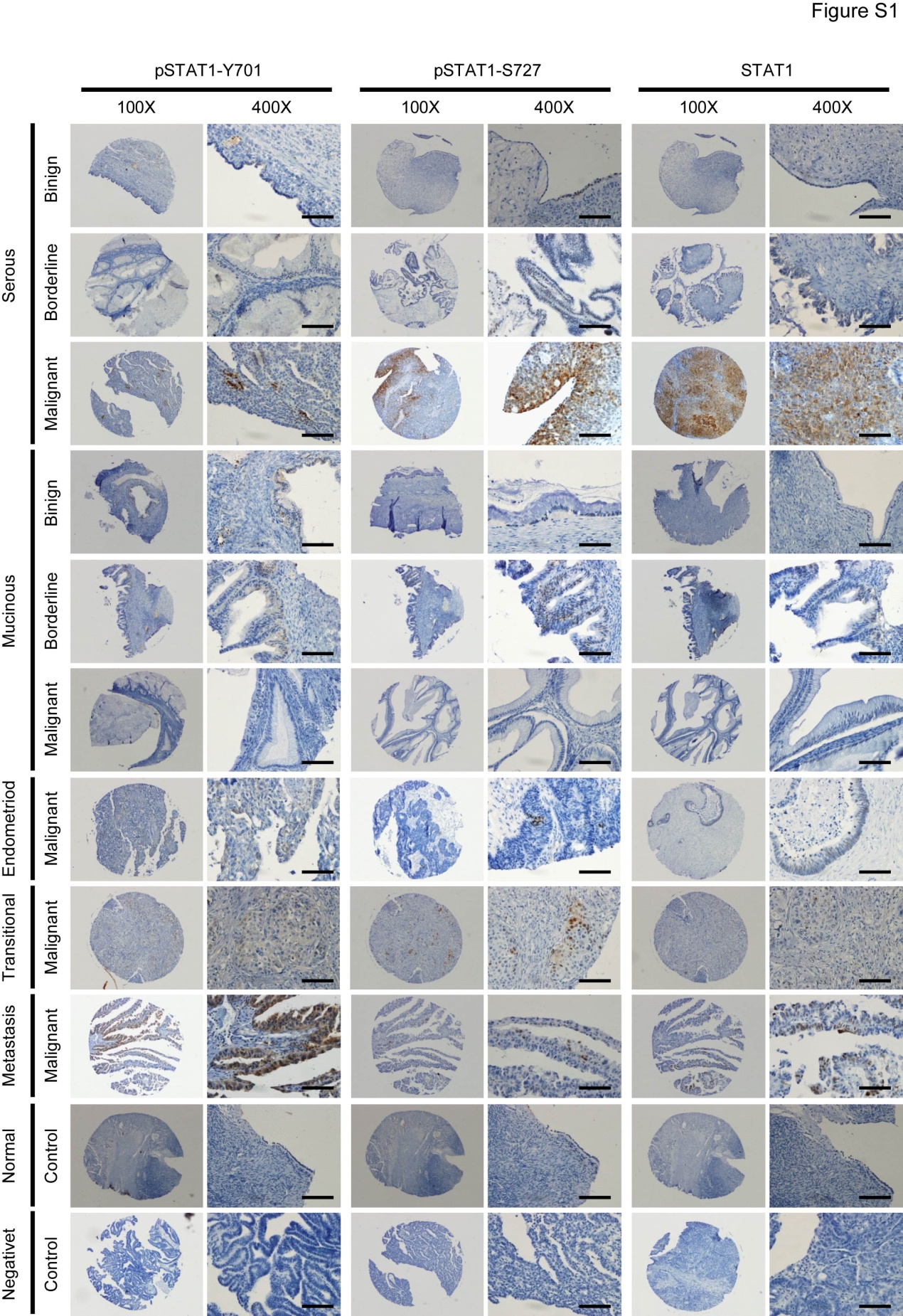
**

**Figure S1** STAT1 expression in human epithelial-type ovarian tumors. Tissue microarray shows the immunohistochemical (IHC) staining of pSTAT1-Y701, pSTAT1-S727, and total STAT1 in serous, mucinous, endometrioid, transitional cell, and metastatic tumors. Normal ovarian tissues were used as a control. IHC without first antibody was used as a negative control. Representative images are shown. A brown color in the epithelial cell is considered as a positive staining. Original magnification x100 and x400; scale bar, 100 µm.


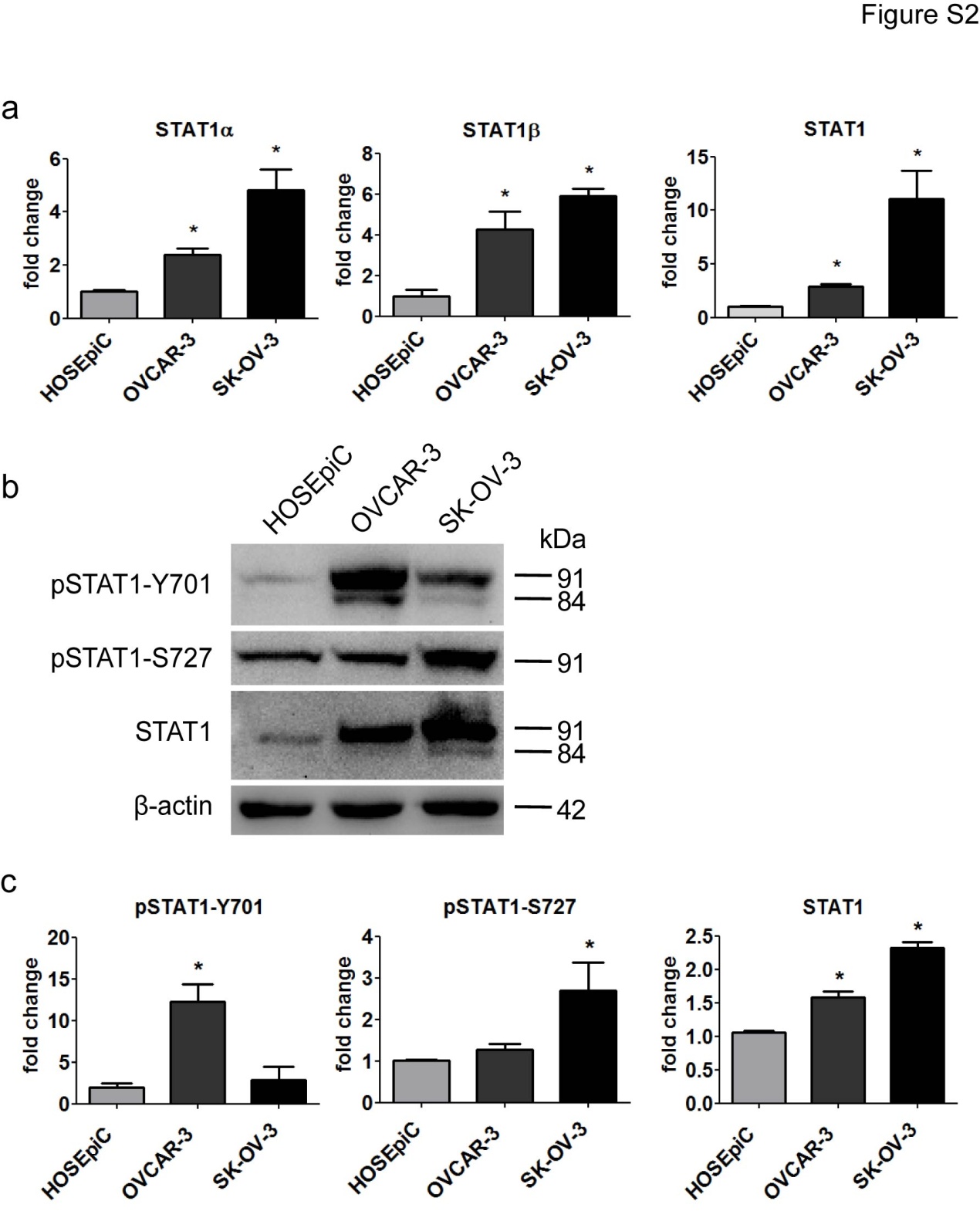


**Figure S2** STAT1 expression in ovarian surface epithelial cells. **a** STAT1 mRNA expression detected by quantitative RT-PCR. The expression of STAT1α, STAT1β, and total STAT1 mRNA was higher in ovarian cancer cells (OVCAR-3 and SK-OV-3) than in non-tumorous control cells (HOSEpiC). **b** STAT1 protein expression detected by immunoblotting. **c** Densitometric analysis of the gels in (**b**). The expression level of total STAT1 was higher in cancer cells (OVCAR-3 and SK-OV-3) than in non-tumorous control cells (HOSEpiC). The highest levels of pSTAT1-Y701 and pSTAT1-S727 were observed in OVCAR-3 and SK-OV-3 cells, respectively, compared with HOSEpiC cells. STAT1α, 91 kDa; STAT1β, 84 kDa; n=3 independent experiments; *, *P* < 0.05 compared to HOSEpiC cells.

**Figure S3** Effect of TGF-β1 on the phosphorylation of STAT1. OVCAR-3 cells were treated with 10 ng/ml of TGF-β1 for 30, 60, or 180 min. TGF-β1 decreased the phosphorylation of STAT1 on Y701, while increased the phosphorylation of STAT1 on S727. INF-γ (10 ng/ml) was used as positive control for the phosphorylation of STAT1 on Y701 and S727. The phosphorylation of Smad2 was increased upon TGF-β1 treatment, whereas the phosphorylation of STAT1 on Y701 and S727 was increased upon INF-γ treatment, indicating the responsiveness of cells to these two cytokines. INF-γ did not alter phospho-Smad2. Neither TGF-β1 nor INF-γ affected total STAT1 and total Smad2 protein expression within this time period.
